# A Note on Gelsemium

**Published:** 1887-07

**Authors:** 


### A NOTE ON GELSEMIUM.

In the April number of the *Southern Journal of Homœopathy*, Dr. Clarence Willard Butler writes of Gelsemium:

"Stimulants afforded prompt relief to persons poisoned with *Gelsem.*, as recorded in Hale's *Materia Medica*, second edition, but this modality seems to have been overlooked in the subsequent arrangement of the symptoms. That many conditions to which *Gelsem.* is curative are relieved by stimulants, notably the lassitude, malaise, all pains except its actively congestive headache, and even its diarrhœas accompanied by colic I have frequently proven clinically."

We are glad to report this important clinical confirmation (of a dubious authority, as we have always regarded Hale's *Materia Medica*) of an useful symptom. We shall be glad to hear further upon the subject from Dr. Butler and others.

